# Mitochondria and Reactive Oxygen Species: The Therapeutic Balance of Powers for Duchenne Muscular Dystrophy

**DOI:** 10.3390/cells13070574

**Published:** 2024-03-26

**Authors:** Silvia Rosanna Casati, Davide Cervia, Paulina Roux-Biejat, Claudia Moscheni, Cristiana Perrotta, Clara De Palma

**Affiliations:** 1 Department of Medical Biotechnology and Translational Medicine (BioMeTra), Università degli Studi di Milano, via Fratelli Cervi 93, 20054 Segrate, Italy; silvia.casati@unimi.it (S.R.C.); clara.depalma@unimi.it (C.D.P.); 2 Department for Innovation in Biological, Agro-Food and Forest Systems (DIBAF), Università degli Studi della Tuscia, Largo dell’Università snc, 01100 Viterbo, Italy; d.cervia@unitus.it; 3 Department of Biomedical and Clinical Sciences (DIBIC), Università degli Studi di Milano, via G.B. Grassi 74, 20157 Milano, Italy; paulina.roux@gmail.com (P.R.-B.); claudia.moscheni@unimi.it (C.M.)

**Keywords:** Duchenne muscular dystrophy, skeletal muscle, mitochondria, mitophagy, oxidative stress, antioxidant defense, redox homeostasis, DMD therapies

## Abstract

Duchenne muscular dystrophy (DMD) is a genetic progressive muscle-wasting disorder that leads to rapid loss of mobility and premature death. The absence of functional dystrophin in DMD patients reduces sarcolemma stiffness and increases contraction damage, triggering a cascade of events leading to muscle cell degeneration, chronic inflammation, and deposition of fibrotic and adipose tissue. Efforts in the last decade have led to the clinical approval of novel drugs for DMD that aim to restore dystrophin function. However, combination therapies able to restore dystrophin expression and target the myriad of cellular events found impaired in dystrophic muscle are desirable. Muscles are higher energy consumers susceptible to mitochondrial defects. Mitochondria generate a significant source of reactive oxygen species (ROS), and they are, in turn, sensitive to proper redox balance. In both DMD patients and animal models there is compelling evidence that mitochondrial impairments have a key role in the failure of energy homeostasis. Here, we highlighted the main aspects of mitochondrial dysfunction and oxidative stress in DMD and discussed the recent findings linked to mitochondria/ROS-targeted molecules as a therapeutic approach. In this respect, dual targeting of both mitochondria and redox homeostasis emerges as a potential clinical option in DMD.

## 1. Introduction

Duchenne muscular dystrophy (DMD) is a genetic progressive muscle-wasting disorder that leads to a rapid loss of mobility and premature death [1]. Its transmission is X-linked recessive and affects almost exclusively young boys; indeed, DMD in females is very rare (<1 per million) and is reported only in individuals with Turner syndrome [2,3,4]. DMD incidence is estimated at ~1 in 5000 newborn boys worldwide [5], characterized by loss-of-function mutations, typically large frameshift deletions, in the DMD gene encoding the dystrophin protein. By contrast, mutations that maintain the reading frame and allow the production of a shorter, but partially functional, dystrophin lead to Becker muscular dystrophy (BMD), a milder form of dystrophinopathy [6].

Dystrophin is a huge subsarcolemmal protein (the full-length muscle isoform, Dp427m, is 427 kDa) with important structural and signaling functions [7]. It is a part of the DAPC (dystrophin-associated protein complex) which is fundamental to connecting the actin cytoskeleton to the extracellular matrix, providing rigidity and integrity to the muscle fiber [8]. The absence of functional dystrophin reduces sarcolemma stiffness and increases fiber susceptibility to contraction damage, triggering a cascade of events ranging from muscle cell degeneration and chronic inflammation to an imbalance between muscle regeneration and degeneration which culminates in an excessive deposition of fibrotic and adipose tissue [7].

The first symptoms of DMD appear in early childhood, around 2–3 years of age, and start to have a strong negative impact on patients’ quality of life. Unfortunately, if not adequately treated, patients die around the age of 30 because of heart or respiratory failure [1]. Despite being primarily a skeletal and cardiac muscle disease, DMD affects other tissues, including the nervous system and bones; thus, most of the patients display cognitive disorders [9,10,11] and scoliosis [12], for instance. Consistently, DMD treatment involves multidisciplinary management that aims to slow down disease progression and alleviate symptoms. Despite the recent advances in translational research and general healthcare, DMD remains 100% fatal. Indeed, clinical strategies for DMD cannot restore muscle tissue and function that is lost; therefore, it is crucial to start the treatment as early as possible. The current DMD therapy can be divided into approaches targeting dystrophin restoration and those aimed at reducing the secondary consequences of dystrophin absence, i.e., a prolonged inflammatory response and the consequent activation of the immune system. The former include the readthrough small agent Ataluren, even if the EMA’s human medicines committee has recently recommended not renewing the marketing authorization obtained in the EU in 2014 [13,14], and exon-skipping antisense oligonucleotides (Eteplirsen [15,16,17], Viltolarsen [18,19,20], Golodirsen [21,22,23], and Casimersen [24]) that have achieved marketing authorization in various jurisdictions starting from 2016. They restore endogenous dystrophin but only in selected subsets of patients. Of note, the FDA approved in 2023 the first gene replacement therapy for DMD (Elevidys), based on AAV-micro-dystrophin gene transfer [25]. On the other side, drugs targeting secondary downstream events include glucocorticoids (Deflazacort and Prednisone) that must be used in patients around 4–5 years of age, but not before 2 years [26,27], and Vamorolone: a novel dissociative anti-inflammatory drug [28].

There is a general agreement that the more muscle quality is maintained, the more dystrophin-restoring therapy will be effective. In this scenario, a variety of innovative approaches and novel pharmacological drugs are being tested in clinical trials, since better treatment options remain a pressing concern. Accordingly, epigenetic drugs such as the histone deacetylase inhibitor Givinostat [29] completed a Phase 3 study and currently, an application to market has been requested by Italfarmaco to the FDA.

Of note, encouraging results have been obtained with the cell-based therapy involving the administration of cardiosphere-derived cells (CAP-1002, Phase 3 clinical trial), which can release extracellular vesicles containing anti-inflammatory and anti-fibrotic molecules [30], and EDG-5506 (Phase 2 clinical trial), a small molecule that inhibits myosin in type II fast-twitch fibers: those that are more susceptible to damage in dystrophic muscle [31].

**Figure 1:**
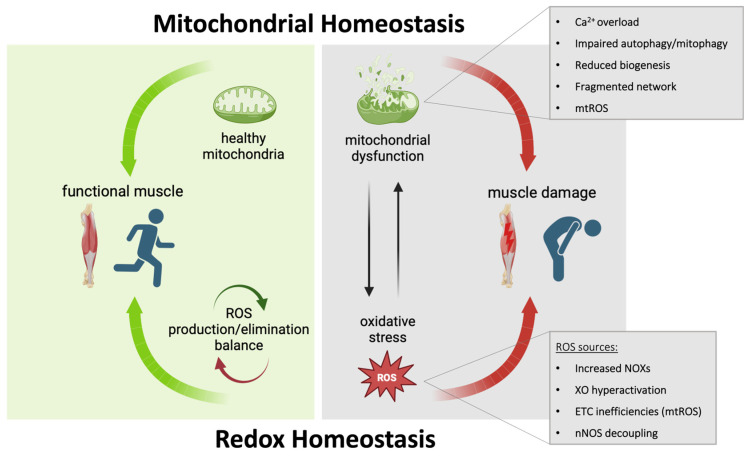
A functional muscle in healthy people is characterized by a proper balance of the redox system and mitochondrial homeostasis (green arrows). In DMD patients, the alteration of this balance contributes to constant muscle damage (red arrows). Many mitochondrial defects have been described in DMD and among these, excessive production of mitochondrial ROS (mtROS) constitutes one of the main sources of oxidative stress in dystrophic muscles, also enhanced by defects in the redox system. Abbreviations: NADPH oxidases (NOXs); xanthine oxidase (XO); electron transport chain (ETC); neuronal nitric oxide synthase (nNOS).

Also, approaches to improve mitochondrial function and reduce oxidative stress, two hallmarks of DMD which are strictly connected, have been recently tested in dystrophinopathies. For instance, Idebenone, an antioxidant drug, induces a lower decline in respiratory function, although only in DMD patients not treated with corticosteroids. Moreover, a Phase 2 clinical trial with Epicatechin, which upregulates peroxisome proliferator-activated receptor-γ co-activator 1, PGC1α, (a member of a family of transcription coactivators that plays a central role in the regulation of cellular energy metabolism) and reduces oxidative stress, is ongoing in BMD patients. Therefore, dual targeting of both mitochondria and redox homeostasis emerges as a potential clinical option for DMD. The purpose of this review is to focus on the role of mitochondrial dysfunction and oxidative stress in the pathogenesis of DMD (Figure 1) and to summarize the recent findings linked to mitochondria/ROS-targeted molecules as a therapeutic approach to treating DMD.

## 2. Mitochondrial Dysfunction in DMD

It is not strange to consider DMD as a metabolic disease, since a strong body of evidence indicates compelling mitochondrial defects in both DMD patients and animal models contributing to the failure of energy homeostasis. Many metabolic pathways are defective, inducing a consistent reduction in resting ATP production [32,33], which reaches sub-threshold levels inconsistent with long-term survival. In DMD, mitochondria work under stress as a result of ADP’s inability to stimulate respiration [34] and in addition, specific defects in complex-I-driven respiration have been reported [35,36]. This occurs even if the energy requirement is high and each of the identified defects has multiple detrimental consequences on the metabolic system; therefore, it is critical to point out a precise defective mechanism. In addition, mitochondrial defects have also been observed in immature dystrophic muscle cells independent of dystrophy deficiency, suggesting intrinsic primary metabolic impairments [37]. Accordingly, cells and tissues from DMD patients with different levels of dystrophin always show mitochondrial deficits, confirming that mitochondria are crucial for the etiology of DMD [32].

Interestingly, a recent study in DMD patients reveals that entire glycolytic, as well as glycogen, pathways are strongly compromised leading to fatty acid accumulation. Also, ADP/ATP cycling and creatine/phosphocreatine shuttling are negatively affected in DMD patients [38]. The citric acid cycle (TCA) is defective due to decreased levels of mitochondrial aconitase (ACO2) as well, while the fatty acid synthase (FASN) that controls lipid synthesis is upregulated, sustaining the accumulation of fatty acid [38].

These effects are strictly associated with dystrophin deficiency; indeed, BMD patients maintain mitochondrial capacity using long-chain fatty acids as the energy source [38] and upregulate l-lactate dehydrogenase B chain (LDHB) and cytosolic malate dehydrogenase (MDH) according to the Warburg effect to keep reasonable ATP levels. Conversely, both enzymes are reduced in DMD resulting in uncontrolled ROS production and limited ATP levels, also sustained by the downregulation of several glycolytic enzymes [38]. This different metabolic rewiring could rely on different fates of substrates; in more severe conditions, such as DMD, an increased competition for substrates may exist redirecting glucose, proteins, or fatty acids to alternative pathways as an adapted response to face larger requests. As reported above, in DMD the biosynthesis of fatty acids increases converting acetyl-CoA, derived from TCA, into fatty acids likely as a result of the high rate of regeneration [33].

In agreement with these metabolic dysfunctions, DMD muscle is unable to adapt to exercise, missing the metabolic changes typically observed in trained muscle, thus contributing to exercise intolerance. In addition, it responds poorly to metabolic enhancers such as 5-aminoimidazole-4-carboxamide ribonucleotide (AICAR) or GW501516 [39,40] whose mainly beneficial effects are associated with utrophin induction. Similarly, metformin, a biguanide that indirectly acts as an AMP-activated protein kinase (AMPK) activator, slightly modulates genes involved in metabolic adaptation, such as those relevant to mitochondrial biogenesis and OxPhos metabolism [41], and its beneficial effects are mostly due to the repression of TGF-β1 signaling [41]. This inability of the dystrophic muscle to cope with energy requests can also be ascribed to epigenetic modifications on the PGC-1α promoter that acquires a compact chromatin structure preventing gene expression. Accordingly, Givinostat restores the PGC-1α promoter’s acetylation, allowing mitochondrial biogenesis and proper metabolic remodeling in DMD muscle [42]. It is emerging that in DMD there are two temporally different phases of mitochondrial impairment, characterized by the initial depletion of mitochondrial mass associated with high autophagic flux but preserved mitochondrial biogenesis. This is followed by autophagy impairment, enabling defective mitochondria accumulation, and progressive inhibition of the biogenesis process, because of epigenetic modifications [42].

Alongside several metabolic defects, mitochondria in *mdx* fibers, one of the murine models of DMD, show dramatic fragmentation. Giacomotto and colleagues [43] report for the first time that mitochondria fragmentation occurs in dystrophic nematodes and a zebrafish model for DMD. They also demonstrate that the repression of drp-1 is beneficial for muscle degeneration. Thereafter, this issue has been studied in mice lacking dystrophin and utrophin (*mdx/Utr−/−*), revealing an imbalance between Drp1 and Mfn2 expression with higher Drp1 levels, confirming an upregulation of fission machinery [44]. Recently, mitochondrial fragmentation has been associated with IP3 receptor (IP3R1) function in a study demonstrating that IP3R1 knockdown modulates mitochondrial dynamics by decreasing the expression of both Drp1 and Fis1 in adult *mdx* fiber [45].

In addition, mitochondria show a Ca^2+^ overload in the matrix and this promotes mitochondrial structural damage over time, especially prolonged permeability transition pore (PTP) opening, resulting in mitochondria depolarization, reduced ATP synthesis, and hydrolysis of glycolytic ATP. In addition, matrix NAD+ is released and respiration is blocked [46,47].

Consistently, preventing PTP opening by cyclosporin A (CsA) or its analog alisporivir, both targeting cyclophilin D whose activity favors PTP opening, improves the *mdx* phenotype, and restores mitochondria activity. However, CsA treatment fails to improve muscle function in DMD patients [48] and exhibits extensive immunosuppressive effects. Even though alisporivir normalizes mitochondria calcium retention and respiration, it suppresses mitochondrial biogenesis, organelle dynamics, and mitophagy in both cardiac and skeletal muscle [49,50]. Otherwise, cyclophilin-D-independent PTP inhibition by TR001 could be a different viable strategy to recover dystrophic muscle damage, fully restoring mitochondrial respiration and membrane potential in both animal models and DMD patients [47].

Along with calcium dysregulation, a remarkable reduction in the efficiency of potassium ion transport has also been identified in *mdx* mice and it is emerging as a potential target to correct mitochondrial dysfunction in DMD. Consistently, NS1619, stimulating a calcium-activated potassium channel (BK_Ca_), restores potassium transport rate and ion content in *mdx* mitochondria, contributing to the improvement of calcium retention capacity and a decrease in oxidative stress [51]. This leads to decreased fibrosis and less muscle degeneration.

**Figure 2:**
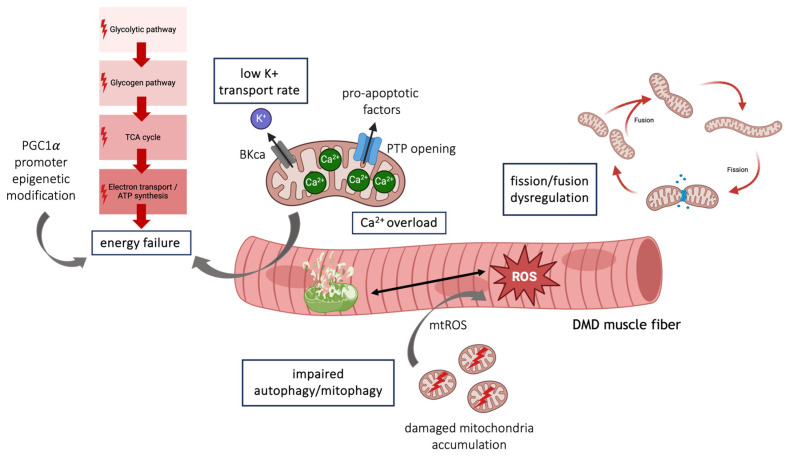
Schematic representation of mitochondrial defects in DMD.

Altogether, this evidence suggests that multi-level mitochondria defects exist in DMD (Figure 2) and this has attracted increasing attention to finding new pharmacological interventions.

### Autophagy and Mitophagy

Damaged mitochondria are removed through mitophagy (selective mitochondria degradation by autophagy) which requires the induction of autophagy and the selective recognition of damaged mitochondria.

Mitophagy is a self-protective mechanism preventing the mitochondrial release of cardiolipin, mitochondrial DNA (mtDNA), and mitochondrial ROS (mtROS) associated with the consequent induction of inflammation and the increase in oxidative stress. Mitophagy is mediated by the PINK1 (PTEN-induced kinase 1)/Parkin (Parkinson juvenile disease protein 2, PARK2) pathway. Normally, PINK1 binds to the outer mitochondrial membrane (OMM) but it is constantly degraded by the presenilin-associated rhomboid-like (PARL) protease of the inner mitochondrial membrane (IMM). In damaged mitochondria, in which the mitochondria membrane potential is lower, PINK1 is not degraded and signals to Parkin, an E3-ubiquitin ligase, labeling mitochondria for degradation.

Many studies have evidenced that defective mitochondria contribute to the pathophysiology of DMD. Mitochondria structure is altered even before the onset of muscle fiber damage and metabolic defects, lowered mitochondrial potential, and promoting excessive mtROS production [52]. In both DMD patients and animal models (mice and worms), autophagy and mitophagy appear to be significantly compromised, leading to the accumulation of damaged mitochondria that could negatively impact the muscle [53,54,55,56]. Interestingly, the inhibition of autophagy is evident with the progression of the disease, concomitant with the fibrotic phase and the exhaustion of stem-cell-mediated regeneration. Conversely, autophagy is active in the early regenerative stage of DMD [56]. This last point is debated and other evidence indicates that autophagy flux is impaired in both young and old *mdx* mice, suggesting that especially in aged dystrophic muscle, lysosomal insufficiency could contribute to failing autophagic flux [57].

The defect is not restricted to muscle; indeed, dystrophic thymocytes also show altered autophagic flux contributing to dysregulated thymocyte differentiation and abnormal T-cell development [58]. Moreover, autophagy defects can be extended to more severe models of DMD, such as golden retriever muscular dystrophy (GRMD) in which impaired autophagy correlates with disease severity [59], and D2-*mdx* mice [60].

Interestingly, DMD autophagy defects have also been associated with gut dysbiosis and a decrease in short-chain fatty acids levels, impairing GPR109A and PPARγ activation. This leads to disinhibition of the endocannabinoid pathway promoting inflammation and autophagy defects [61].

Remarkably, restoring autophagy by a controlled low-protein diet [53], AICAR administration [62], or rapamycin-loaded nanoparticle delivery [63] is an effective strategy to improve dystrophic muscle.

Recent evidence highlights that mitophagy is also compromised in DMD skeletal muscle as witnessed by reduced mitophagy markers in DMD patients even before the onset of symptoms, suggesting that disrupted mitophagy can contribute to DMD pathogenesis.

Mitophagy is also impaired in DMD animal models, in *mdx* mice as well as dystrophic worms, and occurs in mature muscle fiber and muscle stem cells as well [55,64]. Mitophagy dysfunctions are associated with damage-associated molecular patterns (DAMPs) release, such as mtROS and mtDNA, which leads to activation of the NLRP3 inflammasome and promotes IL-1β and IL-18 secretion [54]. Accordingly, enhancing mitophagy by TRIM72 overexpression, a myokine with a protective role in tissue repair and regeneration, blunts the inflammasome increase and mitigates the inflammatory response, with positive effects in DMD [54].

Similarly, urolithin A (UA), a natural microflora-derived metabolite, stimulates mitophagy in DMD models, rescuing mitochondria defects and promoting mitochondrial biogenesis. UA also restores mitophagy in muscle stem cells that regain their regenerative capacity, as well as being able to ameliorate cardiac fibrosis [64]. The positive effects of UA on mitophagy can be associated with increased muscle function in different DMD models, including mice with a more severe phenotype in which UA also prolongs the survival rate.

Alongside skeletal muscles, mitophagy is also defective in the dystrophic heart where, by contrast, autophagy is enhanced [65]. This defect is associated with an altered PINK1/Parkin pathway and accounts for the inability to degrade damaged mitochondria that otherwise accumulate, promoting cardiac dysfunction [65]. Consistently, resveratrol, administered for a long time, stimulates mitophagy in the *mdx* heart, attenuating cardiomyopathy [66].

The positive results obtained by autophagy and mitophagy modulation in dystrophic muscles confirm that the accumulation of damaged mitochondria impacts dystrophic damage and highlights the relevance of both as therapeutic targets.

## 3. Oxidative Stress in DMD

ROS are oxygen-containing free radicals that can chemically react with proteins, DNA, and lipids to modulate their structure and therefore their function [67].

As the skeletal muscle is a highly metabolic tissue, it constantly produces moderate levels of free radicals and is equipped with a sophisticated endogenous antioxidant defense system to ensure tight regulation of redox homeostasis [68,69]. Indeed, at physiological concentrations, free radicals play a fundamental/beneficial role in a myriad of signaling pathways; however, at levels exceeding the hormetic capacity, namely surpassing the buffering capacity of endogenous antioxidants, as in DMD, ROS can cause reversible or irreversible damage [70,71,72,73]. The disruption of redox signaling and control is referred to as oxidative stress [74], which contributes to the pathogenesis of DMD [75]. Elevated oxidative stress, indeed, leads to cell dysfunction and death due to DNA damage, protein oxidation, and lipid peroxidation [76,77]. Therefore, it may exacerbate myofiber damage and necrosis, promote inflammatory cell recruitment to the damaged muscle, and interfere with signaling that can promote repair [77]. Accordingly, oxidative damage has been found to correlate with the severity of muscular dystrophy in human patients [78].

**Figure 3:**
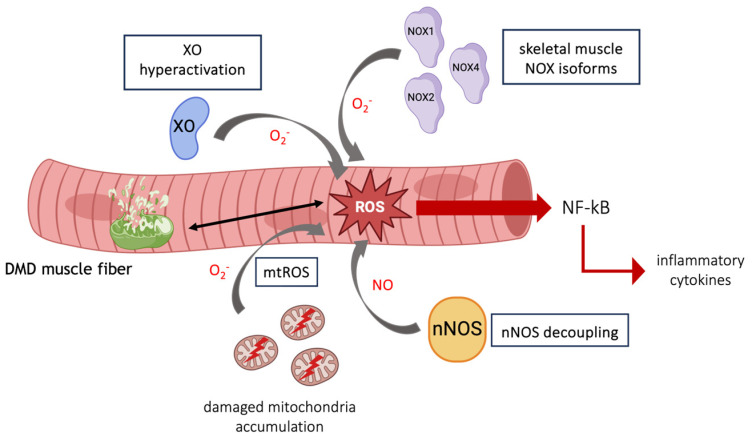
Schematic representation of main sources of free radicals in DMD.

The suspected sources of free radicals in dystrophin deficiency include damaged mitochondria, the activity of nicotinamide adenine dinucleotide phosphate (NADPH) oxidases (NOXs) and xanthine oxidase (XO), expressed either in inflammatory cells or in muscle fibers, and the decoupling of neuronal nitric oxide synthase (nNOS) from the sarcolemma [79,80,81,82] (Figure 3); therefore, drugs acting at these levels have been tested in the treatment of DMD. The mitochondrial electron transport chain and NOXs are currently considered the predominant source of ROS in muscular dystrophy [83,84].

In DMD muscles, the altered production of ROS in damaged mitochondria is a consequence of inefficiencies in the transfer of electrons between the complexes of the electron transport chain (ETC), mainly complexes I and III. The electrons leaked from the respiratory chain react with oxygen, therefore producing ROS [85].

Macrophages and other myeloid cells rapidly invade damaged tissue and generate free radicals to clear the debris; however, the high concentration and non-specificity of ROS-mediated cytolysis aggravate tissue injury. This has been demonstrated in *mdx* mice by the early depletion of macrophages, as well as through the inhibition of key mediators of inflammation such as NF-κB which resulted in reduced cell lysis and improved pathology [86,87,88]. Pro-inflammatory cytokines, such as IL-1β and TNF-α, and oxidants, present in high concentrations in DMD patients, activate the transcription factor NF-κB through the subsequent phosphorylation, polyubiquitination, and proteasomal degradation of the inhibitor protein IκB (I kappa B) [89]. NF-κB then translocates to the nucleus where it binds with DNA and amplifies the generation of pro-inflammatory mediators and ROS notably by regulating NOX activity [90,91,92].

NADPH oxidase is an enzymatic complex utilizing NADPH as a substrate to convert molecular oxygen to ROS, generally superoxide or hydrogen peroxide. Seven isoforms of NOX have been identified and, although NOXs are highly expressed in inflammatory cells, including neutrophils and macrophages, three isoforms are present in the skeletal muscle: NOX1, NOX2, and NOX4 [93]. Increased levels of NOX2 subunits have been found in cultured primary myotubes from *mdx* mice, as well as in their muscle and those of DMD patients, even before the necrotic state, thus before evidence of muscle damage or inflammation [75,84]. During stretched contractions, a pathway involving microtubules and the activation of the microtubule-associated protein rac1 and src kinase stimulates NOX2. The ROS generated by NOX2 further activates src, providing a positive feedback loop, and enhances the influx of calcium, thus leading to increased mitochondrial calcium and additional ROS production [94]. Furthermore, recent evidence suggests that NOX2 activation of src impairs autophagy via the stimulation of the Akt/mTOR pathway and the inhibition of autophagolysosome formation [94]. Interestingly, the pharmacological decrease in NOX2 obtained with the administration of simvastatin improved the *mdx* phenotype by enhancing diaphragm force and reducing fibrosis. These functional improvements were accompanied by autophagy activation and the decline of oxidative stress [95].

NOX4 is predominantly expressed in cardiac myocytes and increased levels of NOX4 have been long noted in dystrophic mice [81]. This has been recently confirmed in human DMD iPSC-derived cardiomyocytes, where elevated ROS production from hyperactive NOX4 in the mitochondria contributes to cell death [96]. NOX4 upregulation has been revealed in the muscle of D2.*mdx* mice, a severe mouse model of DMD, where it has also been shown that inhibiting NOX4 reduces muscle fibrosis, therefore promoting the beneficial remodeling of diseased muscles [97].

XO, which generates superoxide, is hyperactive in dystrophin-deficient muscle of *mdx* mice and DMD patients and contributes to muscular dysfunction. XO can be stimulated by ischemia, aberrant calcium homeostasis, and disrupted DAPC, all of which are features of DMD [98]. The attenuation of XO-induced superoxide production by the allopurinol metabolite, oxypurinol, preserved the drop in the eccentric contraction force typical of DMD [98].

Muscles of dystrophic mice and dogs also exhibit elevated activity of myeloperoxidase (MPO), an enzyme that is mainly expressed by neutrophils and to a lesser extent by monocytes and macrophages in the inflamed area. MPO catalyzes the production of ROS, especially the highly reactive hypochlorous acid from hydrogen peroxide, which increases oxidative stress and boosts muscle cell lysis [99,100,101].

### The Key Role of the Antioxidant Defense System in Dystrophic Skeletal Muscle

The endogenous antioxidant defense system can work directly by scavenging ROS through the modulation of glutathione (GSH), superoxide dismutase (SOD), catalase (CAT) levels (Phase 1), or indirectly by inducing the cytoprotective (Phase 2) response, i.e., heme oxygenase 1 (HO-1), NAD(P)H:quinone acceptor oxidoreductase 1 (NQO1), and glutamate cysteine ligase (GCL), predominately regulated by the transcription factor NF-E2-related factor 2 (Nrf2) [102]. However, in the context of DMD, the exacerbated production of free radicals overwhelms the already defective endogenous antioxidant defense system [103,104].

Initially, the oxidative damage in DMD was attributed to the flawed GSH system. GSH is the most abundant low-molecular-weight thiol, and GSH/glutathione disulfide (GSSG) is a crucial cellular redox couple. Under physiological conditions, GSH is generated by de novo synthesis via a two-step process requiring the enzymes GCL and glutathione synthetase, while GSH levels are maintained by the NADPH-dependent enzyme glutathione reductase which recycles GSSG [105]. In DMD, the total GSH is reduced by 50% due to the decreased activity of GCL, the rate-limiting enzyme in GSH synthesis [78]. Moreover, the increased activity of GSH metabolizing enzymes, e.g., glutathione peroxidase and glutathione reductase, with the contemporary increase in the GSSG/GSH ratio being evidence of the hyperoxidative status observed in dystrophic muscles [106].

While the GSH/GSSG ratio has been widely used as an indicator of oxidative stress [107], a recent study on DMD patients has proposed dynamic thiol-disulfide homeostasis as a novel marker of oxidative stress in the disease [108]. The level of thiols, a class of organic compounds containing a sulfhydryl group (SH), including glutathione, may be considered a valuable antioxidant parameter, as in the presence of oxidants they can form reversible disulfide (SS) bonds. Also, dystrophic animal models present an increase in protein thiol oxidation which reflects the extent of reversible oxidation [109,110]. The treatment of *mdx* mice with the antioxidant compound n-acetyl-cysteine (NAC) that targets thiol oxidation decreased reversible protein thiol oxidation [111] and had benefits against the disease by improving the morphology of limb and diaphragm muscles as well as the heart, decreasing the levels of TNF-α, and increasing muscle strength [82,111,112,113]. These beneficial effects may be due to the ability of NAC to act as an antioxidant, but also to the increase in intracellular glutathione levels through increasing the levels of cellular cysteine [114]. However, NAC administration was accompanied by a significant reduction in body and muscle weight in *mdx* mice [115], generating concerns regarding its use in patients. The question is debated since it has been recently demonstrated that NAC decreases the abnormal fiber branching responsible for *mdx* fiber hypertrophy, thus preventing the increase in muscle mass characteristic of the dystrophinopathies [116].

The Nrf2 signaling pathway plays a pivotal role in oxidative stress and inflammation [117,118]. Under normal conditions, Nrf2 is kept inactive by being bound to its cytoplasmic endogenous inhibitor Keap1, which promotes its ubiquitination and degradation. Upon exposure to stress signals such as free radicals, the conformational change in Keap1 induces its dissociation from Nrf2 which then translocates to the nucleus. There, Nrf2 binds to antioxidant-related elements (ARE) and modulates the expression of a multitude of defensive genes, including those encoding for SO, CAT, NQO1, and HO-1. The Nrf2 pathway is activated in dystrophic patients to counteract oxidative stress [75].

Notably, being associated with the regulation of over 600 target genes, the Nrf2 signaling pathway is also involved in prolonging satellite cell proliferation, calcium handling, mitohormesis, autophagy, and heat shock proteins [119]. Due to its ability to tackle simultaneously numerous cellular processes, Nrf2 is a particularly interesting therapeutical target for the multisystemic disorder DMD [120]. Antioxidant compounds, such as curcumin, and sulforaphane have been shown to activate Nrf2 and be effective in the treatment of preclinical models of DMD, by alleviating dystrophic muscle pathology [117,118,121]. Very recently, dimethyl fumarate (DMF), a small molecule that acts through the activation of Nrf2, has been tested in *mdx* mice in a short-term study, showing pro-mitochondrial effects, improving the histopathology, and augmenting muscle performance [122]. Other antioxidant enzymes of the Nrf2 pathway have been investigated in DMD as possible targets of pharmacological treatment. A major class of enzymatic antioxidants is SOD. Three isoforms of SOD exist (SOD1, 2, 3) with different cellular localization: SOD1 is in the cytosol and the mitochondrial intermembrane space, SOD2 is in the mitochondrial matrix, and SOD3 in the extracellular space [104]. Both SOD1 and SOD2 expression is significantly increased in the muscles of dystrophic patients [75]. This, however, may have rather a detrimental than a protective role, as its mechanism consists in converting superoxide to hydrogen peroxide and can cause lipid peroxidation [123]; supporting this idea, the overexpression of SOD1 in transgenic mice increased the levels of lipid peroxidation in the muscle cytosolic proteins [123]. This might be one explanation for the early unsuccessful clinical trials of antioxidants for DMD patients, as a superoxide dismutase mimetic was attempted [124].

Catalase is a key enzyme of cellular oxidative balance and cellular redox signaling regulation, as it hydrolyzes the highly reactive hydrogen peroxide (H_2_O_2_) into water and O2 [125]; therefore, it has been hypothesized that its overexpression would result in improved dystrophic skeletal muscle. Indeed, the overexpression of CAT improved the muscle function of *mdx* mice [126], as well as the cardiomyocyte function of DMD iPSC-derived cardiomyocytes [127]. HO-1 is responsible for the synthesis of biliverdin and bilirubin, which are non-enzymatic antioxidants present in skeletal muscle. HO-1 is at the core of Nrf2-mediated NF-κB inhibition by catalyzing the degradation of heme into carbon monoxide (CO), Fe^2+^, and biliverdin which is consequently reduced to the antioxidant bilirubin [128]. In DMD, low levels of HO-1 coupled with high concentrations of IL-6, exacerbate inflammation [75]. Moreover, pharmacological inhibition or genetic ablation of HO-1 worsens muscular dystrophy while, in contrast, pharmacological induction of HO-1 improves the phenotype of *mdx* mice [128,129].

Although DMD is a genetic disease, oxidative stress is a central mediator of its multifaceted pathogenesis and not only a deleterious secondary process accompanying tissue damage due to the inflammatory response.

The inconsistent results obtained in the early attempts to counteract oxidative stress point out the complexity of redox imbalance in DMD muscles. Fortunately, the extensive research and technological advancements of the last decades aimed at identifying ROS/RNS sources have paved the way to develop treatments targeting specific players in the oxidative stress and antioxidant defense system of dystrophic muscle.

## 4. Clinical Trials with Mitochondria/ROS-Targeted Drugs

In the last decades, many efforts have been made at the preclinical and clinical levels to develop therapeutic strategies for DMD targeting mitochondria, including lifestyle interventions (dietary supplements) and pharmacological treatments. These strategies have been mainly focused on reducing mitochondrial dysfunction and oxidative stress, also maintaining mitochondrial quality/quantity in DMD.

At the time of this review, 391 clinical trials regarding Duchenne and Becker muscular dystrophy have been registered in the US (https://www.clinicaltrials.gov/ct2/results?cond=Duchenne+Muscular+Dystrophy&term=&cntry=&state=&city=&dist= (accessed on 18 February 2024)) and 106 in Europe (https://www.clinicaltrialsregister.eu/ctr-search/search?query=Duchenne+muscular+dystrophy (accessed on 18 February 2024)), including both open and closed studies. Many of these studies were interventional (clinical trials) envisaging a pharmacological treatment. A list of the compounds currently tested in clinical trials is reported in Table 1.

One of the most pursued approaches in DMD is to promote oxidative metabolism in skeletal muscle and in this regard, one beneficial target is PPARδ which increases fatty acid oxidation, sparing glucose use and greatly improving energy production [130]. One molecule, ASP0367 (bocidelpar sulfate), is in a Phase 1b clinical trial to assess safety, tolerability, and preliminary efficacy in DMD boys (NCT04184882), and a previous Phase 1 study has been conducted on healthy adults demonstrating that the drug is well-tolerated, rapidly absorbed, and modulates PPARδ target genes [131].

Energy metabolism is also fostered by (-)-epicatechin, which is a flavonol, a type of flavonoid, acting as an exercise mimetic and a potent inducer of mitochondrial biogenesis in both skeletal and heart muscle [132,133]. It also upregulates follistatin with positive effects on muscle growth, inflammation, and fibrosis [134], and a Phase 1 study (NCT04386304) is ongoing to assess the safety and efficacy in Becker patients.

A drug extensively used in DMD to positively modulate energy metabolism is resveratrol. Resveratrol is a polyphenolic extract of red wine, also found in grape skins. It activates the SIRT1-PGC-1α axis and has antioxidant proprieties; it also acts as an antifibrotic agent on skeletal and cardiac muscle and can reduce cardiac hypertrophy [135,136,137,138]. Resveratrol has been administered to DMD patients showing benefits such as reduced CK levels and enhanced motor function; however, the number of patients enrolled in the study is low (only five for DMD) [139].

Another promising approach is the repurposing of an antidiabetic drug. Metformin is a biguanide with several mechanisms of action, which include the activation of AMPK associated with the inhibition of respiratory chain complex I and the ability to upregulate nNOS, which is defective in DMD. Based on this, metformin has been combined with l-citrulline, a nitric oxide (NO) precursor. In both Becker and DMD conditions, the combination showed promising results [140,141], but recently a randomized double-blind clinical trial on 47 ambulant DMD patients revealed that overall l-citrulline plus metformin did not reduce the motor function decline [142]. However, positive outcomes have been observed only in a subgroup of stable patients with a steady muscle performance decline [142], indicating high intraindividual variability. This limited the power of the study, even if the results in the more homogeneous subgroup are supportive of combination therapy for the treatment of DMD patients.

Among mitochondria-targeting agents, we can also ascribe Givinostat, a pan-HDACi that promotes muscle regeneration and reduces inflammation and fibrosis. However, its epigenetic mechanism of action is crucial to unlocking the mitochondrial biogenesis program [42], increasing oxidative metabolism. In a Phase 2 study (NCT01761292), Givinostat showed good safety and tolerability associated with histological improvements in DMD muscles [29]. Currently, a Phase 3 clinical trial has been completed to evaluate the long-term efficacy of the drug in ambulant DMD patients (NCT02851797) and an application to market has been requested for Givinostat in DMD.

## 5. Conclusions

Efforts in the last decade have led to the clinical approval of novel therapy products for DMD that aim to restore dystrophin function, with other approvals likely soon [31]. We are learning lessons from these drug development programs that will have a big impact on the DMD field and, more generally, on molecular and cellular biology. On the other hand, there is a general agreement that a degree of personalization will be required to address the diversity of genetic defects causing DMD and that better therapies are still needed, applicable to a wider group of patients. Thus, combination therapies able to restore dystrophin expression and target the myriad of cellular events found impaired in dystrophic muscle are desirable. Indeed, in patients with established pathology, the restoration of dystrophin might be insufficient since the progressive decline in muscle quality is due to chronic inflammation and fibro/fatty degeneration. In this scenario, the knowledge gained on the role of mitochondrial (dys)function and redox balance in the pathogenesis of DMD might pave the way for new drug discovery efforts with maximum benefit to patients. For instance, mitochondria/ROS-targeted molecules to simultaneously improve mitochondrial function and reduce ROS accumulation seem to have a key potential for the ongoing development of DMD therapies but will also be highly useful in the development of treatments for other diseases sharing similar pathological mechanisms.

## Figures and Tables

**Table 1:** Compounds treating mitochondrial dysfunction/oxidative stress currently in clinical trials.

Compound	Molecular/Cellular Target	Clinical Trial Stage	Registration Number	References
ASP0367	PPARδ	Phase 1b	NCT04184882	[130,131]
(-)-epicatechin	Energy metabolism	Phase 1	NCT04386304	[132,133,134]
resveratrol	SIRT1-PGC-1α axis/oxidative stress	Phase 2a	UMIN000014836	[135,136,137,138,139]
metformin	AMPK/respiratory chain complex I	Phase 2 Phase 3	NCT02018731 NCT01995032	[140,141,142]
l-citrulline	NO system	Phase 2 Phase 3	NCT02018731 NCT01995032	[140,141,142]
givinostat	HDAC/mitochondrial biogenesis program	Phase 2 Phase 3	NCT01761292 NCT02851797	[29,42]
